# Supplementary figures and images for: Artificial liver research output and citations from 2004 to 2017: a bibliometric analysis
Source: PeerJ. 2019 Jan 11;6:e6178. doi: 10.7717/peerj.6178 (PMC6330953; doi:10.7717/peerj.6178)

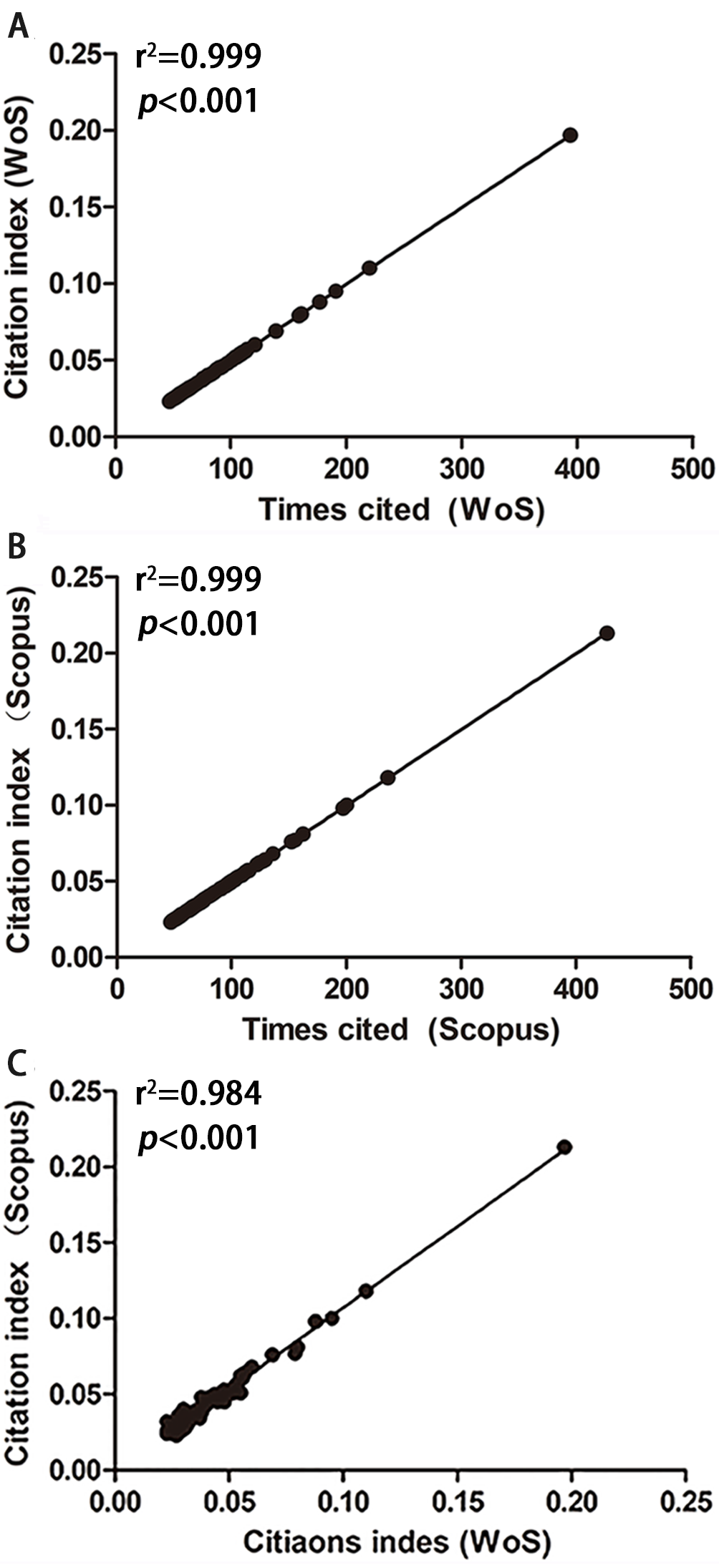

Supplement: Supplemental Information 1 — The relation between citation index and the number of citations respectively in WoS(A) and Scopus(B), and the citation index between WoS and Scopus(C). [file peerj-07-6178-s001.png]

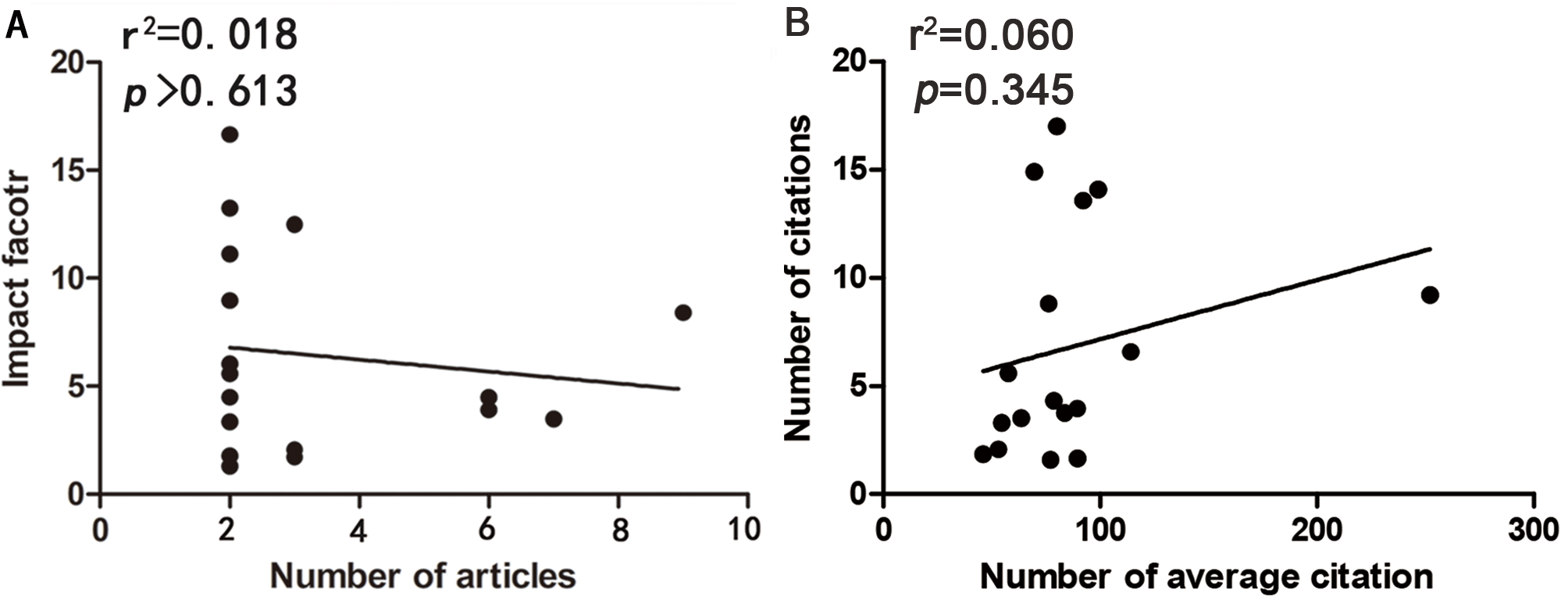

Supplement: Supplemental Information 2 — The relation between the number of articles and the impact of factors in T18 journals (A), and between the number of citations and the impact of factors in T18 journals (B). [file peerj-07-6178-s002.png]

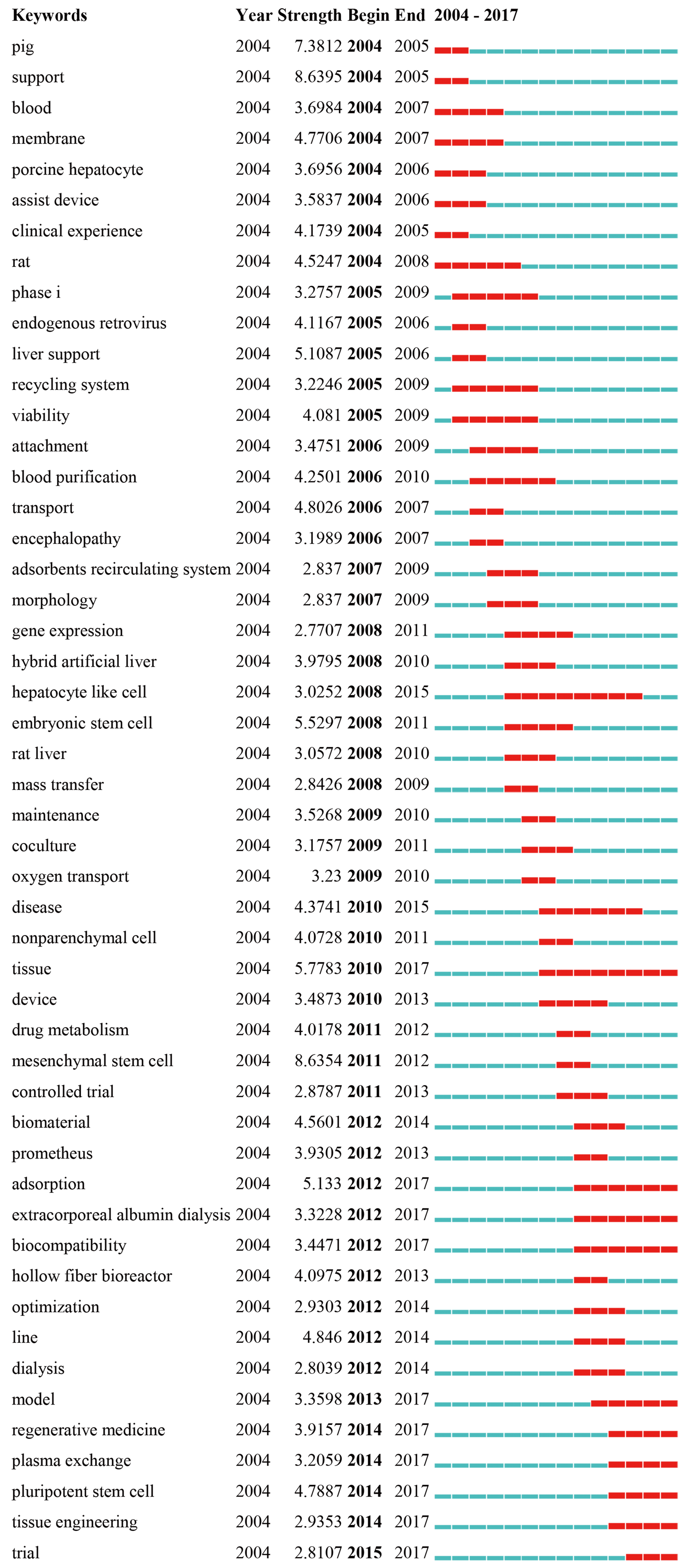

Supplement: Supplemental Information 3 [file peerj-07-6178-s003.png]
