# Supplementary material for: Artificial liver research output and citations from 2004 to 2017: a bibliometric analysis
Source: PeerJ. 2019 Jan 11;6:e6178. doi: 10.7717/peerj.6178 (PMC6330953; doi:10.7717/peerj.6178)
Supplement: Supplemental Information 4 [file peerj-07-6178-s004.docx]

| **Rank** | **Articles** | **Publication year** | **Times cited (Web)** | **Citation index**  **(Web)** | **Times cited (Scopus)** | **Citaitons index (Scopus)** |
| --- | --- | --- | --- | --- | --- | --- |
| 1 | Demetriou A A, Jr B R, Busuttil R W, et al. Prospective, randomized, multicenter, controlled trial of a bioartificial liver in treating acute liver failure. [J]. Annals of Surgery, 2016, 239(5):667-70. | 2004 | 394 | 0.197 | 427 | 0.213 |
| 2 | Lee P J, Hung P J, Lee L P. An artificial liver sinusoid with a microfluidic endothelial‐like barrier for primary hepatocyte culture[J]. Biotechnology & Bioengineering, 2007, 97(5):1340-6. | 2007 | 220 | 0.110 | 236 | 0.118 |
| 3 | Kelm J M, Fussenegger M. Microscale tissue engineering using gravity-enforced cell assembly[J]. Trends in Biotechnology, 2004, 22(4):195-202. | 2004 | 191 | 0.095 | 200 | 0.100 |
| 4 | Medical applications of membranes: Drug delivery, artificial organs and tissue engineering | 2008 | 177 | 0.088 | 197 | 0.098 |
| 5 | Stamatialis D F, Papenburg B J, Gironés M, et al. Medical applications of membranes: Drug delivery, artificial organs and tissue engineering[J]. Journal of Membrane Science, 2008, 308(1–2):1-34. | 2006 | 161 | 0.080 | 162 | 0.081 |
| 6 | Huang P, Zhang L, Gao Y, et al. Direct Reprogramming of Human Fibroblasts to Functional and Expandable Hepatocytes[J]. Cell Stem Cell, 2014, 14(3):370-384. | 2014 | 159 | 0.079 | 155 | 0.077 |
| 7 | Kim B S, Park I K, Hoshiba T, et al. Design of artificial extracellular matrices for tissue engineering[J]. Progress in Polymer Science, 2011, 36(2):238-268. | 2011 | 139 | 0.069 | 152 | 0.076 |
| 8 | Khuroo M S, Khuroo M S, Farahat K L. Molecular adsorbent recirculating system for acute and acute-on-chronic liver failure: a meta-analysis[J]. Liver Transpl, 2004, 10(9):1099-1106. | 2004 | 121 | 0.060 | 136 | 0.068 |
| 9 | Fukuda J, Sakai Y, Nakazawa K. Novel hepatocyte culture system developed using microfabrication and collagen/polyethylene glycol microcontact printing[J]. Biomaterials, 2006, 27(7):1061-70. | 2006 | 114 | 0.057 | 129 | 0.064 |
| 10 | Yamada M, Utoh R, Ohashi K, et al. Controlled formation of heterotypic hepatic micro-organoids in anisotropic hydrogel microfibers for long-term preservation of liver-specific functions[J]. Biomaterials, 2012, 33(33):8304-8315. | 2012 | 113 | 0.056 | 122 | 0.061 |
| 11 | Olson J C, Wendon J A, Kramer D J, et al. Intensive care of the patient with cirrhosis. [J]. Hepatology, 2011, 54(5):1864-72. | 2011 | 113 | 0.056 | 127 | 0.063 |
| 12 | Park J K, Lee D H. Bioartificial liver systems: current status and future perspective[J]. Journal of Bioscience & Bioengineering, 2005, 99(4):311-319. | 2005 | 111 | 0.055 | 102 | 0.051 |
| 13 | Mp V D K, Hoekstra R, Chamuleau R A, et al. Clinical application of bioartificial liver support systems. [J]. Annals of Surgery, 2004, 240(2):216. | 2004 | 110 | 0.055 | 122 | 0.061 |
| 14 | Kulig K M, Vacanti J P. Hepatic tissue engineering[J]. Transplant Immunology, 2004, 12(3–4):303-310. | 2004 | 110 | 0.055 | 124 | 0.062 |
| 15 | Carpentier B, Gautier A, Legallais C. Artificial and bioartificial liver devices: present and future[J]. Gut, 2009, 58(12):1690-1702. | 2009 | 109 | 0.054 | 115 | 0.057 |
| 16 | Shan J, Schwartz R E, Ross N T, et al. Identification of small molecules for human hepatocyte expansion and iPS differentiation[J]. Nature Chemical Biology, 2013, 9(8):514-520. | 2013 | 108 | 0.054 | 114 | 0.057 |
| 17 | Volpi N, Schiller J, Stern R, et al. Role, metabolism, chemical modifications and applications of hyaluronan[J]. Current Medicinal Chemistry, 2009, 16(14):-. | 2009 | 107 | 0.053 | 112 | 0.056 |
| 18 | Carraro A, Hsu W M, Kulig K M, et al. In vitro analysis of a hepatic device with intrinsic microvascular-based channels[J]. Biomedical Microdevices, 2008, 10(6):795-805. | 2008 | 105 | 0.052 | 109 | 0.054 |
| 19 | Nahmias Y, Berthiaume F, Yarmush M L. Integration of technologies for hepatic tissue engineering. [J]. Adv Biochem Eng Biotechnol, 2006, 103:309-329. | 2007 | 104 | 0.052 | 103 | 0.051 |
| 20 | Park J, Berthiaume F, Toner M, et al. Microfabricated grooved substrates as platforms for bioartificial liver reactors. [J]. Biotechnology & Bioengineering, 2005, 90(5):632-44. | 2005 | 101 | 0.050 | 104 | 0.052 |
| 21 | Shupe T, Williams M, Brown A, et al. Method for the decellularization of intact rat liver. [J]. Organogenesis, 2010, 6(2):134-136. | 2010 | 97 | 0.048 | 98 | 0.049 |
| 22 | Wang S, Nagrath D, Chen P C, et al. Three-Dimensional Primary Hepatocyte Culture in Synthetic Self-Assembling Peptide Hydrogel[J]. Tissue Engineering Part A, 2008, 14(2):227-36. | 2008 | 97 | 0.048 | 106 | 0.053 |
| 23 | Park H, Cannizzaro C, Vunjak-Novakovic G, et al. Nanofabrication and microfabrication of functional materials for tissue engineering[J]. Tissue Engineering, 2007, 13(8):1867. | 2007 | 97 | 0.048 | 90 | 0.045 |
| 24 | Seo S J, Kim I Y, Choi Y J, et al. Enhanced liver functions of hepatocytes cocultured with NIH 3T3 in the alginate/galactosylated chitosan scaffold[J]. Biomaterials, 2006, 27(8):1487-1495. | 2006 | 97 | 0.048 | 97 | 0.048 |
| 25 | Wang X, Yan Y, Zhang R. Rapid prototyping as a tool for manufacturing bioartificial livers[J]. Trends in Biotechnology, 2007, 25(11):505. | 2007 | 93 | 0.046 | 99 | 0.049 |
| 26 | Chen A A, Thomas D K, Ong L L, et al. Humanized mice with ectopic artificial liver tissues[J]. Proc Natl Acad Sci U S A, 2011, 108(29):11842-11847. | 2011 | 91 | 0.045 | 95 | 0.047 |
| 27 | Wong S F, No d Y, Choi Y Y, et al. Concave microwell based size-controllable hepatosphere as a three-dimensional liver tissue model. [J]. Biomaterials, 2011, 32(32):8087-8096. | 2011 | 90 | 0.045 | 91 | 0.045 |
| 28 | Barakat O, Abbasi S, Rodriguez G, et al. Use of Decellularized Porcine Liver for Engineering Humanized Liver Organ[J]. Journal of Surgical Research, 2012, 173(1): e11. | 2012 | 88 | 0.044 | 99 | 0.049 |
| 29 | Peter K, Bernd H, Rudolf S, et al. In vivo quantification of liver dialysis: comparison of albumin dialysis and fractionated plasma separation. [J]. Digest of the World Core Medical Journals, 2006, 43(3):451-457. | 2005 | 88 | 0.044 | 100 | 0.050 |
| 30 | Brophy C M, Luebke-Wheeler J L, Amiot B P, et al. Rat hepatocyte spheroids formed by rocked technique maintain differentiated hepatocyte gene expression and function. [J]. Hepatology, 2009, 49(2):578. | 2009 | 85 | 0.042 | 92 | 0.046 |
| 31 | Yan Y, Wang X, Xiong Z, et al. Direct Construction of a Three-dimensional Structure with Cells and Hydrogel[J]. Journal of Bioactive & Compatible Polymers, 2005, 20(3):259-269. | 2005 | 83 | 0.041 | 96 | 0.048 |
| 32 | Chan C, Berthiaume F, Nath B D, et al. Hepatic tissue engineering for adjunct and temporary liver support: critical technologies. [J]. Liver Transplantation, 2004, 10(11):1331–1342. | 2004 | 81 | 0.040 | 95 | 0.047 |
| 33 | Fiegel H C, Kaufmann P M, Bruns H, et al. Hepatic tissue engineering: from transplantation to customized cell-based liver directed therapies from the laboratory[J]. Journal of Cellular & Molecular Medicine, 2008, 12(1):56–66. | 2008 | 80 | 0.040 | 86 | 0.043 |
| 34 | Bleiziffer O, Eriksson E, Yao F, et al. Gene transfer strategies in tissue engineering[J]. Journal of Cellular & Molecular Medicine, 2007, 11(2):206-223. | 2007 | 77 | 0.038 | 82 | 0.041 |
| 35 | Nyberg S L, Hardin J, Amiot B, et al. Rapid, large-scale formation of porcine hepatocyte spheroids in a novel spheroid reservoir bioartificial liver[J]. Liver Transpl, 2005, 11(8):901-910. | 2005 | 77 | 0.038 | 84 | 0.042 |
| 36 | Haque T, Chen H, Ouyang W, et al. In vitro study of alginate-chitosan microcapsules: an alternative to liver cell transplants for the treatment of liver failure[J]. Biotechnology Letters, 2005, 27(5):317-322. | 2005 | 77 | 0.038 | 76 | 0.038 |
| 37 | Schwartz R E, Linehan J L, Painschab M S, et al. Defined conditions for development of functional hepatic cells from human embryonic stem cells. [J]. Stem Cells & Development, 2005, 14(6):643-655. | 2005 | 76 | 0.038 | 97 | 0.048 |
| 38 | Saliba F, Camus C, Durand F, et al. Albumin dialysis with a noncell artificial liver support device in patients with acute liver failure: a randomized, controlled trial. [J]. Annals of Internal Medicine, 2013, 159(8):522-31. | 2013 | 75 | 0.037 | 80 | 0.040 |
| 39 | Chamuleau R A, Deurholt T, Hoekstra R. Which are the right cells to be used in a bioartificial liver? [J]. Metabolic Brain Disease, 2005, 20(4):327-335. | 2005 | 75 | 0.037 | 69 | 0.034 |
| 40 | Li S, Xiong Z, Wang X, et al. Direct Fabrication of a Hybrid Cell/Hydrogel Construct by a Double-nozzle Assembling Technology[J]. Journal of Bioactive & Compatible Polymers, 2009, 24(3):249-265. | 2009 | 71 | 0.035 | 78 | 0.039 |
| 41 | Fukuda J, Nakazawa K. Orderly arrangement of hepatocyte spheroids on a microfabricated chip[J]. Tissue Engineering, 2005, 11(8):1254-1262. | 2005 | 71 | 0.035 | 71 | 0.035 |
| 42 | Larsen F S, Schmidt L E, Bernsmeier C, et al. High-volume plasma exchange in patients with acute liver failure: An open randomised controlled trial. [J]. Journal of Hepatology, 2016, 64(1):69-78. | 2016 | 69 | 0.034 | 75 | 0.037 |
| 43 | Selden C, Hodgson H. Cellular therapies for liver replacement[J]. Transplant Immunology, 2004, 12(3–4):273-288. | 2004 | 69 | 0.034 | 74 | 0.037 |
| 44 | Imamura T, Cui L, Teng R, et al. Embryonic stem cell-derived embryoid bodies in three-dimensional culture system form hepatocyte-like cells in vitro and in vivo[J]. Tissue Engineering Part A, 2004, 10(12):1716-1724. | 2004 | 67 | 0.033 | 72 | 0.036 |
| 45 | Mckenzie T J, Lillegard J B, Nyberg S L. Artificial and bioartificial liver support.[J]. Seminars in Liver Disease, 2008, 28(02):210-217. | 2008 | 65 | 0.032 | N/A | N/A |
| 46 | Mazza G, Rombouts K, Hall A R, et al. Decellularized human liver as a natural 3D-scaffold for liver bioengineering and transplantation[J]. Scientific Reports, 2015, 5:13079. | 2015 | 64 | 0.032 | 71 | 0.035 |
| 47 | Mei J, Sgroi A, Mai G, et al. Improved survival of fulminant liver failure by transplantation of microencapsulated cryopreserved porcine hepatocytes in mice[J]. Cell Transplantation, 2009, 18(1):101. | 2009 | 64 | 0.032 | 63 | 0.031 |
| 48 | Lee K H, Shin S J, Kim C B, et al. Microfluidic synthesis of pure chitosan microfibers for bio-artificial liver chip.[J]. Lab on A Chip, 2010, 10(10):1328. | 2010 | 63 | 0.031 | 67 | 0.033 |
| 49 | Sen S, Rose C, Ytrebã¸ L M, et al. Effect of albumin dialysis on intracranial pressure increase in pigs with acute liver failure: a randomized study[J]. Critical Care Medicine, 2006, 34(1):158-164. | 2006 | 63 | 0.031 | 66 | 0.033 |
| 50 | Walkup M H, Gerber D A. Hepatic stem cells: in search of.[J]. Stem Cells, 2006, 24(8):1833-1840. | 2006 | 63 | 0.031 | 57 | 0.028 |
| 51 | Ran F, Nie S, Li J, et al. Heparin-like macromolecules for the modification of anticoagulant biomaterials[J]. Macromolecular Bioscience, 2012, 12(1):116-125. | 2012 | 62 | 0.031 | 57 | 0.028 |
| 52 | Park J, Li Y, Berthiaume F, et al. Radial flow hepatocyte bioreactor using stacked microfabricated grooved substrates[J]. Biotechnology & Bioengineering, 2008, 99(2):455-467. | 2008 | 62 | 0.031 | 56 | 0.028 |
| 53 | Dan Y Y, Yeoh G C. Liver stem cells: A scientific and clinical perspective[J]. Journal of Gastroenterology & Hepatology, 2008, 23(5):687-698. | 2008 | 62 | 0.031 | 56 | 0.028 |
| 54 | Yu Y, Fisher J E, Lillegard J B, et al. Cell therapies for liver diseases.[J]. Liver Transplantation, 2012, 18(1):9–21. | 2012 | 61 | 0.030 | 55 | 0.027 |
| 55 | Martin Y, Eldardiri M, Lawrencewatt D J, et al. Microcarriers and their potential in tissue regeneration.[J]. Tissue Eng Part B Rev, 2011, 17(1):71-80. | 2011 | 61 | 0.030 | 64 | 0.032 |
| 56 | Chu X H, Shi X L, Feng Z Q, et al. Chitosan nanofiber scaffold enhances hepatocyte adhesion and function[J]. Biotechnology Letters, 2009, 31(3):347. | 2009 | 61 | 0.030 | 54 | 0.027 |
| 57 | Sen S, Williams R, Jalan R. Emerging indications for albumin dialysis.[J]. American Journal of Gastroenterology, 2005, 100(2):468-75. | 2005 | 61 | 0.030 | 66 | 0.033 |
| 58 | Wang X, Yan Y, Zhang R. Recent trends and challenges in complex organ manufacturing[J]. Tissue Eng Part B Rev, 2010, 16(2):189-197. | 2010 | 60 | 0.030 | 80 | 0.040 |
| 59 | Bartolo L D, Salerno S, Curcio E, et al. Human hepatocyte functions in a crossed hollow fiber membrane bioreactor[J]. Biomaterials, 2009, 30(13):2531-43. | 2009 | 60 | 0.030 | 65 | 0.032 |
| 60 | Hay D C, Pernagallo S, Diazmochon J J, et al. Unbiased screening of polymer libraries to define novel substrates for functional hepatocytes with inducible drug metabolism. [J]. Stem Cell Research, 2011, 6(2):92-102. | 2011 | 59 | 0.029 | 62 | 0.031 |
| 61 | Hu Y, Haynes M T, Wang Y, et al. A Highly Efficient Synthetic Vector: Non-Hydrodynamic Delivery of DNA to Hepatocyte Nuclei In Vivo[J]. Acs Nano, 2013, 7(6):5376-84. | 2013 | 58 | 0.029 | 56 | 0.028 |
| 62 | Bhatia S N, Underhill G H, Zaret K S, et al. Cell and tissue engineering for liver disease. [J]. Science Translational Medicine, 2014, 6(245):245sr2. | 2014 | 57 | 0.028 | 73 | 0.036 |
| 63 | Struecker B, Raschzok N, Sauer I M. Liver support strategies: cutting-edge technologies.[J]. Nature Reviews Gastroenterology & Hepatology, 2014, 11(3):166. | 2014 | 57 | 0.028 | 65 | 0.032 |
| 64 | Anne Weber †, Groyer-Picard M T, Franco D, et al. Hepatocyte transplantation in animal models[J]. Liver Transplantation, 2009, 15(1):7–14. | 2009 | 57 | 0.028 | 53 | 0.026 |
| 65 | Yi N J, Suh K S, Lee H W, et al. An artificial vascular graft is a useful interpositional material for drainage of the right anterior section in living donor liver transplantation[J]. Liver Transplantation, 2007, 13(8):1159. | 2007 | 57 | 0.028 | 56 | 0.028 |
| 66 | Detry O, Roover A D, Honoré P, et al. Brain edema and intracranial hypertension in fulminant hepatic failure: Pathophysiology and management[J]. World Journal of Gastroenterology, 2006, 12(46):7405-7412. | 2006 | 57 | 0.028 | 68 | 0.034 |
| 67 | Rozga J. Liver support technology – an update[J]. Xenotransplantation, 2006, 13(5):380-389. | 2006 | 57 | 0.028 | 64 | 0.032 |
| 68 | Seo S J, Choi Y J, Akaike T, et al. Alginate/galactosylated chitosan/heparin scaffold as a new synthetic extracellular matrix for hepatocytes[J]. Tissue Engineering Part A, 2006, 12(1):33-44. | 2006 | 56 | 0.028 | 60 | 0.030 |
| 69 | Kataoka K, Nagao Y, Nukui T, et al. An organic–inorganic hybrid scaffold for the culture of HepG2 cells in a bioreactor[J]. Biomaterials, 2005, 26(15):2509. | 2005 | 56 | 0.028 | 57 | 0.028 |
| 70 | Du W B, Li L J, Huang J R, et al. Effects of artificial liver support system on patients with acute or chronic liver failure. [J]. Transplantation Proceedings, 2005, 37(10):4359-4364. | 2005 | 56 | 0.028 | 51 | 0.025 |
| 71 | Dalgetty D M, Medine C N, Iredale J P, et al. Progress and future challenges in stem cell-derived liver technologies[J]. Am J Physiol Gastrointest Liver Physiol, 2009, 297(2):241-8. | 2009 | 55 | 0.027 | 62 | 0.031 |
| 72 | Zinchenko Y S, Schrum L W, Clemens M, et al. Hepatocyte and kupffer cells co-cultured on micropatterned surfaces to optimize hepatocyte function. [J]. Tissue Engineering Part A, 2006, 12(4):751-761. | 2006 | 55 | 0.027 | 53 | 0.026 |
| 73 | Ebrahimkhani M R, Neiman J A, Raredon M S, et al. Bioreactor technologies to support liver function in vitro. [J]. Adv Drug Deliv Rev, 2014, 69-70:132-157. | 2014 | 54 | 0.027 | 63 | 0.031 |
| 74 | Zhang M Y, Lee P J, Hung P J, et al. Microfluidic environment for high density hepatocyte culture[J]. Biomedical Microdevices, 2008, 10(1):117-121. | 2008 | 54 | 0.027 | 50 | 0.025 |
| 75 | Gu H Y, Chen Z, Sa R X, et al. The immobilization of hepatocytes on 24 nm-sized gold colloid for enhanced hepatocytes proliferation[J]. Biomaterials, 2004, 25(17):3445-3451. | 2004 | 54 | 0.027 | 47 | 0.023 |
| 76 | Miki T, Ring A, Gerlach J. Hepatic differentiation of human embryonic stem cells is promoted by three-dimensional dynamic perfusion culture conditions. [J]. Tissue Engineering Part C Methods, 2011, 17(5):557. | 2011 | 53 | 0.026 | 56 | 0.028 |
| 77 | Cho C H, Park J, Tilles A W, et al. Layered patterning of hepatocytes in co-culture systems using microfabricated stencils[J]. Biotechniques, 2010, 48(1):47-52. | 2010 | 53 | 0.026 | 60 | 0.030 |
| 78 | De B L, Salerno S, Morelli S, et al. Long-term maintenance of human hepatocytes in oxygen-permeable membrane bioreactor[J]. Biomaterials, 2006, 27(27):4794-4803. | 2006 | 53 | 0.026 | 55 | 0.027 |
| 79 | Forbes S J, Newsome P N. New horizons for stem cell therapy in liver disease. [J]. Journal of Hepatology, 2012, 56(2):496-9. | 2012 | 52 | 0.026 | 51 | 0.025 |
| 80 | Tostões R M, Leite S B, Miranda J P, et al. Perfusion of 3D encapsulated hepatocytes--a synergistic effect enhancing long-term functionality in bioreactors. [J]. Biotechnology & Bioengineering, 2015, 108(1):41-49. | 2011 | 52 | 0.026 | 57 | 0.028 |
| 81 | Stéphenne X, Najimi M, Sokal E M. Hepatocyte cryopreservation:Is it time to change the strategy?[J]. World Journal of Gastroenterology, 2010, 16(1):1. | 2010 | 52 | 0.026 | 53 | 0.026 |
| 82 | Linke K, Schanz J, Hansmann J, et al. Engineered liver-like tissue on a capillarized matrix for applied research[J]. Tissue Engineering, 2007, 13(11):2699. | 2007 | 52 | 0.026 | 54 | 0.027 |
| 83 | Ogawa S, Tagawa Y, Kamiyoshi A, et al. Crucial Roles of Mesodermal Cell Lineages in a Murine Embryonic Stem Cell–Derived In Vitro Liver Organogenesis System[J]. Stem Cells, 2010, 23(7):903-913. | 2005 | 52 | 0.026 | 51 | 0.025 |
| 84 | Nie C, Ma L, Xia Y, et al. Novel heparin-mimicking polymer brush grafted carbon nanotube/PES composite membranes for safe and efficient blood purification[J]. Journal of Membrane Science, 2015, 475(475):455-468. | 2015 | 51 | 0.025 | 50 | 0.025 |
| 85 | Baddour J A, Sousounis K, Tsonis P A. Organ repair and regeneration: an overview[J]. Birth Defects Research Part C Embryo Today Reviews, 2012, 96(1):1-29. | 2012 | 51 | 0.025 | 57 | 0.028 |
| 86 | Schanz J, Pusch J, Hansmann J, et al. Vascularised human tissue models: a new approach for the refinement of biomedical research[J]. Journal of Biotechnology, 2010, 148(1):56-63. | 2010 | 51 | 0.025 | 53 | 0.026 |
| 87 | Mavridamelin D, Damelin L H, Eaton S, et al. Cells for bioartificial liver devices: the human hepatoma-derived cell line C3A produces urea but does not detoxify ammonia. [J]. Biotechnology & Bioengineering, 2008, 99(3):644-51. | 2008 | 51 | 0.025 | 54 | 0.027 |
| 88 | Wang X, Yan Y, Lin F, et al. Preparation and characterization of a collagen/chitosan/heparin matrix for an implantable bioartificial liver[J]. J Biomater Sci Polym Ed, 2005, 16(9):1063-1080. | 2005 | 51 | 0.025 | 50 | 0.025 |
| 89 | Jalan R, Sen S, Williams R. Prospects for extracorporeal liver support[J]. Gut, 2004, 53(6):890-898. | 2004 | 51 | 0.025 | 54 | 0.027 |
| 90 | Wu, Xu-Bo; Tao, Ran. Hepatocyte differentiation of mesenchymal stem cells[J]. HEPATOBILIARY & PANCREATIC DISEASES INTERNATIONAL, 2012, 11(4): 360-371. | 2012 | 50 | 0.025 | 50 | 0.025 |
| 91 | Cho C H, Parashurama N, Park E Y, et al. Homogeneous differentiation of hepatocyte-like cells from embryonic stem cells: applications for the treatment of liver failure. [J]. Faseb Journal Official Publication of the Federation of American Societies for Experimental Biology, 2008, 22(3):898. | 2008 | 50 | 0.025 | 52 | 0.026 |
| 92 | Maguire T, Davidovich A E, Wallenstein E J, et al. Control of hepatic differentiation via cellular aggregation in an alginate microenvironment[J]. Biotechnology & Bioengineering, 2007, 98(3):631-44. | 2007 | 50 | 0.025 | 52 | 0.026 |
| 93 | Du C, Narayanan K, Leong M F, et al. Induced pluripotent stem cell-derived hepatocytes and endothelial cells in multi-component hydrogel fibers for liver tissue engineering. [J]. Biomaterials, 2014, 35(23):6006. | 2014 | 49 | 0.024 | 55 | 0.027 |
| 94 | Calandrelli L, Calarco A, Laurienzo P, et al. Compatibilized polymer blends based on PDLLA and PCL for application in bioartificial liver[J]. Biomacromolecules, 2008, 9(6):1527-1534. | 2008 | 49 | 0.024 | 50 | 0.025 |
| 95 | El B A, Kizner L, Schueler V, et al. First use of the Molecular Adsorbent Recirculating System technique on patients with hypoxic liver failure after cardiogenic shock. [J]. Asaio Journal, 2004, 50(4):332. | 2004 | 49 | 0.024 | 57 | 0.028 |
| 96 | Stutchfield B M, Forbes S J, Wigmore S J. Prospects for stem cell transplantation in the treatment of hepatic disease. [J]. Liver Transpl, 2010, 16(7):827–836. | 2010 | 48 | 0.024 | 49 | 0.024 |
| 97 | Stutchfield B M, Simpson K, Wigmore S J. Systematic review and meta-analysis of survival following extracorporeal liver support[J]. Br J Surg, 2011, 98(5):623-631. | 2011 | 47 | 0.023 | 48 | 0.024 |
| 98 | Hoganson D M, Vacanti J P. Tissue engineering and organ structure: a vascularized approach to liver and lung[J]. Pediatric Research, 2008, 63(5):520-6. | 2008 | 47 | 0.023 | 52 | 0.026 |
| 99 | Krisper P, Stauber R E. Technology Insight: artificial extracorporeal liver support—how does Prometheus® compare with MARS[J]. Nat Clin Pract Nephrol, 2007, 3(5):267-276. | 2007 | 47 | 0.023 | 64 | 0.032 |
| 100 | Fiegel HC; Havers J; Kneser U; Smith MK; Moeller T; Kluth D; Mooney DJ; Rogiers X; Kaufmann PM. Influence of flow conditions and matrix coatings on growth and differentiation of three-dimensionally cultured rat hepatocytes. [J]. Tissue Engineering, 2004, 10(2):165-174. | 2004 | 47 | 0.023 | 49 | 0.024 |
