# Supplementary material for: Artificial liver research output and citations from 2004 to 2017: a bibliometric analysis
Source: PeerJ. 2019 Jan 11;6:e6178. doi: 10.7717/peerj.6178 (PMC6330953; doi:10.7717/peerj.6178)
Supplement: Supplemental Information 5 [file peerj-07-6178-s005.docx]

**Additional file 4** Keyword ranked by the number of citations on artificial livers from 2004 to 2017

| **keyword** | **year** | **Centrality** | **Count** |
| --- | --- | --- | --- |
| bioartificial liver | 2004 | 0.61 | 405 |
| hepatocyte | 2004 | 0.25 | 248 |
| in vitro | 2004 | 0.05 | 172 |
| transplantation | 2004 | 0.12 | 151 |
| fulminant hepatic failure | 2004 | 0.06 | 132 |
| culture | 2004 | 0.09 | 124 |
| failure | 2004 | 0 | 112 |
| acute liver failure | 2004 | 0.05 | 108 |
| bioreactor | 2004 | 0.11 | 108 |
| support | 2004 | 0.07 | 99 |
| cell | 2004 | 0.03 | 96 |
| stem cell | 2004 | 0.03 | 80 |
| differentiation | 2004 | 0.09 | 80 |
| rat hepatocyte | 2004 | 0 | 78 |
| liver failure | 2004 | 0.05 | 77 |
| system | 2004 | 0 | 76 |
| controlled trial | 2004 | 0.04 | 74 |
| albumin dialysis | 2004 | 0.21 | 73 |
| liver | 2004 | 0 | 73 |
| support system | 2004 | 0.01 | 72 |
| extracellular matrix | 2004 | 0.01 | 67 |
| artificial liver | 2004 | 0 | 65 |
| tissue engineering | 2004 | 0 | 59 |
| hepatic failure | 2004 | 0.02 | 59 |
| expression | 2004 | 0 | 53 |
| spheroid | 2004 | 0.05 | 53 |
| gene expression | 2004 | 0 | 51 |
| scaffold | 2005 | 0.02 | 50 |
| assist device | 2004 | 0.01 | 49 |
| cirrhosis | 2004 | 0.08 | 47 |
| adsorbent recirculating system | 2005 | 0.03 | 46 |
| porcine hepatocyte | 2004 | 0 | 46 |
| artificial liver support | 2004 | 0.01 | 42 |
| embryonic stem cell | 2004 | 0.03 | 42 |
| mar | 2004 | 0.08 | 41 |
| Bioartificial liver | 2004 | 0 | 39 |
| tissue | 2004 | 0 | 38 |
| model | 2005 | 0 | 38 |
| growth factor | 2004 | 0 | 37 |
| membrane | 2004 | 0 | 36 |
| hepatocyte transplantation | 2004 | 0 | 36 |
| rat | 2004 | 0 | 36 |
| regeneration | 2004 | 0 | 35 |
| rat liver | 2004 | 0 | 34 |
| pig | 2004 | 0 | 33 |
| therapy | 2004 | 0 | 33 |
| experience | 2004 | 0.04 | 32 |
| cryopreservation | 2004 | 0.03 | 32 |
| adult rat hepatocyte | 2004 | 0.04 | 32 |
| liver transplantation | 2004 | 0 | 32 |
| mesenchymal stem cell | 2008 | 0 | 32 |
| survival | 2004 | 0 | 32 |
